# Modeling the Presence of Myelin and Edema in the Brain Based on Multi-Parametric Quantitative MRI

**DOI:** 10.3389/fneur.2016.00016

**Published:** 2016-02-17

**Authors:** Marcel Warntjes, Maria Engström, Anders Tisell, Peter Lundberg

**Affiliations:** ^1^Center for Medical Image Science and Visualization (CMIV), Linköping University, Linköping, Sweden; ^2^Division of Cardiovascular Medicine, Department of Medical and Health Sciences, Linköping University, Linköping, Sweden; ^3^Radiology, Department of Medical and Health Sciences, Linköping University, Linköping, Sweden; ^4^Radiation Physics, Department of Medical and Health Sciences, Linköping University, Linköping, Sweden

**Keywords:** quantitative magnetic resonance imaging, brain tissue modeling, myelin, edema, T_1_ relaxation, T_2_ relaxation, proton density

## Abstract

The aim of this study was to present a model that uses multi-parametric quantitative MRI to estimate the presence of myelin and edema in the brain. The model relates simultaneous measurement of R_1_ and R_2_ relaxation rates and proton density to four partial volume compartments, consisting of myelin partial volume, cellular partial volume, free water partial volume, and excess parenchymal water partial volume. The model parameters were obtained using spatially normalized brain images of a group of 20 healthy controls. The pathological brain was modeled in terms of the reduction of myelin content and presence of excess parenchymal water, which indicates the degree of edema. The method was tested on spatially normalized brain images of a group of 20 age-matched multiple sclerosis (MS) patients. Clear differences were observed with respect to the healthy controls: the MS group had a 79 mL smaller brain volume (1069 vs. 1148 mL), a 38 mL smaller myelin volume (119 vs. 157 mL), and a 21 mL larger excess parenchymal water volume (78 vs. 57 mL). Template regions of interest of various brain structures indicated that the myelin partial volume in the MS group was 1.6 ± 1.5% lower for gray matter (GM) structures and 2.8 ± 1.0% lower for white matter (WM) structures. The excess parenchymal water partial volume was 9 ± 10% larger for GM and 5 ± 2% larger for WM. Manually placed ROIs indicated that the results using the template ROIs may have suffered from loss of anatomical detail due to the spatial normalization process. Examples of the application of the method on high-resolution images are provided for three individual subjects: a 45-year-old healthy subject, a 72-year-old healthy subject, and a 45-year-old MS patient. The observed results agreed with the expected behavior considering both age and disease. In conclusion, the proposed model may provide clinically important parameters, such as the total brain volume, degree of myelination, and degree of edema, based on a single qMRI acquisition with a clinically acceptable scan time.

## Introduction

Myelin is crucial for efficient signal transmission over long ranges in the nervous system because it increases the speed at which the impulses propagate along the axons. Axons are coated piecewise by multiple layers of phospholipid membranes (“sheaths”) with embedded proteins produced by oligodendrocytes and Schwann cells in the central and peripheral nervous systems, respectively. Degradation of myelin impairs the signal transmission, and the nerve may eventually wither, leading to brain atrophy and brain dysfunction. Knowledge of myelin content supports the investigation of early brain development (1, 2). Accurate myelin measurements are valuable in studies of neurodegenerative diseases, such as multiple sclerosis (MS) (3, 4) and dementia (5–7). Thus, measurements and monitoring of myelin content would provide important information for the diagnosis and prognosis in patients with suspected myelin degradation.

One established MRI method for myelin detection is based on the measurement of the multi-exponential transverse T_2_ relaxation time using a Carr–Purcell–Meiboom–Gill (CPMG) sequence (8–10). The short-time component of the observed T_2_ relaxation represents the presence of water trapped between the myelin sheaths, termed myelin water (MyW), whereas the medium-time T_2_ relaxation component is attributed to the intra- and extracellular water. Commonly, the myelin water fraction (MWF), corresponding to the ratio of both components, is calculated. The proportionality of MWF with the myelin content has been verified *in vitro* and by histopathology (11, 12). More recently, an alternative approach called mcDESPOT was developed (13). This method consists of a combination of spoiled gradient echo (SPGR) and balanced steady-state free precession (bSSFP) acquisitions at multiple flip angles, resulting in the measurement of MyW and intra- and extracellular water pools. In particular, the mcDESPOT method has been applied to myelin development in children (14).

Limitations of the two described methods are mainly practical. Due to the very short myelin T_2_ relaxation time (10–15 ms), the multi-exponent T_2_ measurement mainly depends on the amplitude of the first echo signal, and mcDESPOT is highly sensitive to the accuracy of the applied flip angle, making the measurements demanding in terms of both SNR and time as well as highly dependent on corrections for B_1_ field and RF pulse profile effects. The underlying models of both approaches are considerably different, resulting in widespread estimations of the myelin content.

Here, we propose a model to estimate the presence of myelin and edema in the brain based on multi-parametric quantitative MRI (qMRI), where the longitudinal relaxation rate R_1_, transverse relaxation rate R_2_, and proton density PD are determined simultaneously in one acquisition. It was previously reported that pathological processes, such as axonal damage, gliosis, inflammation, and edema are related to changes in the values of R_1_, R_2_, and PD (15–19). Currently, multi-parametric MR quantification of R_1_, R_2_, and PD can be achieved at high resolution within a 6–8 min scan time (20), which would make such an approach attractive for routine clinical use. The aim of this study was to present a model that relates the appearance of a qMRI-derived R_1_–R_2_–PD data structure to the myelin partial volume of the brain. The model parameters were derived based on data from Ref. (21), where brain images of a group of healthy controls were spatially normalized and averaged to characterize the healthy brain. The second aim of this study was to explore the possibilities of the model to detect both the differences in myelin content and the presence of edema in the pathological brain. Examples of the application of the method are provided for a group of MS patients and three individual subjects.

## Materials and Methods

### The Relaxation Model

**Figure 1:**
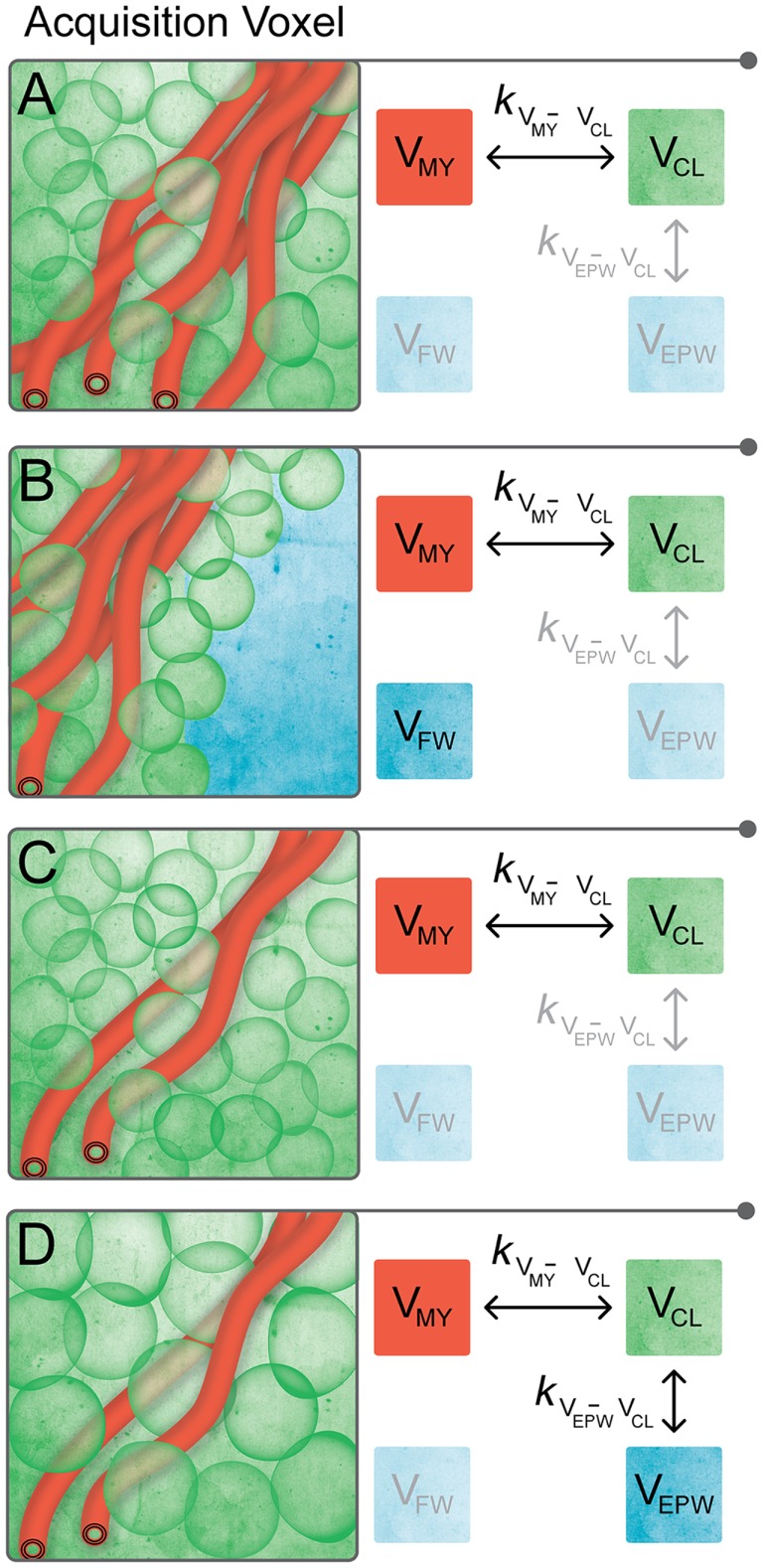
**Proposed compartmental exchange system for modeling brain parenchyma**. Each MRI acquisition voxel is composed of four partial volume compartments, where each partial volume can range from 0 to 100%, and where the sum is 100%. A compartment is grayed out when its partial volume is equal to 0. **(A)** Normal brain parenchyma consists of myelin partial volume V_MY_ and cellular partial volume V_CL_. Between V_MY_ and V_CL_, there is a magnetization net exchange rate *k*_VMY-VCL_. **(B)** At the interface of brain parenchyma with the surrounding bulk CSF, an acquisition voxel contains a mixture of V_MY_ and V_CL_ (i.e., brain parenchyma) and free water partial volume V_FW_. There is no magnetization exchange between V_FW_ and the other partial volumes. **(C)** In pathological brain parenchyma, myelin loss may occur, resulting in a relative decrease in V_MY_. The relative amount of V_CL_ in the acquisition voxel increases to maintain 100% tissue, resulting in a decrease in the total brain volume. **(D)** Alternatively, there can be edema in pathological brain parenchyma, included in the model by the presence of the non-zero excess parenchymal water partial volume V_EPW_. No distinction can be made between excess parenchymal water and the already present parenchymal water of the V_CL_, making the exchange rate *k*_VEPW-VCL_ infinitely high. The combination of V_CL_ and V_EPW_ effectively dilutes the myelin content, resulting in a relative decrease in V_MY_ per acquisition voxel and an increase in the total brain volume.

The proposed model for the observed R_1_, R_2_, and PD values of the brain is visualized in Figure 1: each MRI acquisition voxel is composed of four partial volume compartments: the myelin partial volume V_MY_, cellular partial volume V_CL_, free water partial volume V_FW_, and excess parenchymal water partial volume V_EPW_. The content in each partial volume compartment can range from 0 to 100%, where the sum of the four compartments is 100%. Each partial volume compartment has its own relaxation properties (R_1,MY_, R_2,MY_, PD_MY_, R_1,CL_, R_2,CL_, PD_CL_, R_1,FW_, R_2,FW_, PD_FW_, R_1,EPW_, R_2,EPW_, PD_EPW_), without further detailed knowledge of the multitude of interacting pools within each of the compartments. Using this approach, each partial volume compartment can be described by its R_1_–R_2_–PD values, its fraction of the acquisition voxel and the magnetization exchange with other partial volume compartments. The total acquisition voxel exhibits R_1_–R_2_–PD values that reflect the effective, combined relaxation behavior of all four compartments. An MR quantification sequence measures the effective R_1_–R_2_–PD values of acquisition voxels in the total imaging volume, which can provide input to the model.

The V_MY_ contains the thin layers of MyW and myelin sheets that are closely packed around the axons. The close proximity of MyW to the surrounding structure results in a very fast relaxation of this compartment. The V_CL_ consists of intra- and extracellular (interstitial) water, axonal water, and all cellular macromolecules, not being related to myelin. The presence of the macromolecules results in a medium-time relaxation of V_CL_, which is slower than V_MY_, but longer than V_FW_. Between V_MY_ and V_CL_, there is a magnetization exchange rate *k*_VMY-VCL_. In the model, acquisition voxels in the normal brain parenchyma contain a mixture of V_MY_ and V_CL_, where voxels in gray matter (GM) have a low V_MY_ and voxels in white matter (WM) have a high V_MY_ (see Figure 1A). The two compartments V_MY_ and V_CL_ are an approximation of the four-pool model (22), where V_MY_ contains MyW and myelin semi-solids and V_CL_ contains intracellular and extracellular water and non-myelin semi-solids pools, albeit with less degrees of freedom.

The brain is surrounded by cerebrospinal fluid (CSF), making it necessary to add a free water partial volume V_FW_ to the model, as also pointed out in Ref. (23). Because bulk CSF is physically separated from the brain parenchyma except for the interface, there is no magnetization exchange between V_FW_ and any other compartment (i.e. “free”). Hence, at the border of the brain, acquisition voxels contain a mixture of V_MY_ and V_CL_ (brain parenchyma) and V_FW_ (CSF), see Figure 1B.

In the pathological brain, two distinct processes are modeled: compared with the normal brain, there can be myelin loss, resulting in a relative decrease in V_MY_. To maintain 100% tissue, the relative amount of V_CL_ in an acquisition voxel will increase. Therefore, the loss of myelin results in a compaction of the brain and, thus, a decrease in the total brain volume (Figure 1C). The second process is the occurrence of edema, modeled as the presence of excess parenchymal water partial volume V_EPW_, which adds water to V_CL_. No distinction can be made between excess parenchymal water and the already present parenchymal water of V_CL_ and, therefore, the exchange rate *k*_VEPW-VCL_ is infinitely high. Modeling two separate partial volume compartments with an infinite exchange is a mathematical approach to acquire knowledge on the degree of edema without knowledge of the exact internal composition of V_CL_. The cellular swelling due to a non-zero V_EPW_ effectively dilutes the myelin present in the acquisition voxel, resulting in a relative decrease in V_MY_. In this case, the total brain volume increases (Figure 1D).

### Bloch Simulation

A numerical simulation of coupled Bloch equations of the four partial volume compartments was performed using 150 identical magnetization elements *i*, spread equidistantly over a distance of 15 mm in the acquisition slice direction, where each element had a distance ${\text{d}}_{\text{i}}$ from the center of the slice. Each of the 150 elements consisted of the same partial volume distribution of interacting V_MY_, V_CL_, V_FW_, and V_EPW_ with normalized magnetization vectors *M*_MY_, *M*_CL_, *M*_FW_, and *M*_EPW_, respectively. The evolution of each magnetization $M_{\text{i}}={\left[\begin{array}{ccc} M_{\text{x}} & M_{\text{y}} & M_{\text{z}} \end{array}\right]}_{i}^{\text{T}}$ was calculated in small time-steps *t*, where each sequential magnetization ${\text{M}}_{\text{i,n}+\text{1}}$ of each element *i* was calculated from the original magnetization *M*_i,n_ using:

(1) $$M_{i,n+1}={\text{R}}_{\mathrm{RF}}*{\text{R}}_{\mathrm{GR}}*{\text{R}}_{R1}*{\text{R}}_{R2}*M_{i,n}$$

***R***_RF_ is the rotation matrix for the applied slice-selective RF pulses. The envelope of the RF pulses was approximated by a series of block pulses with constant amplitudes over the time interval *t*. The rotation flip angle α, achieved in time *t* over the x- or y-axis, is equal to 2πγ${\text{B}}_{\text{1}}$*t*, where B_1_ is the amplitude of the RF pulse at that particular time interval, and γ is the gyromagnetic ratio. ***R***_GR_ is the rotation matrix for the applied slice-selective gradients. The rotation flip angle ω, achieved in time *t* over the z-axis, is equal to 2πγ${\text{Gd}}_{\text{i}}\text{t}$, where *G* is the gradient strength and ${\text{d}}_{\text{i}}$ is the distance from the center of the slice.

***R***_R1_ is the relaxation matrix for the elements for the longitudinal relaxation rate R_1_. ***R***_R1_ only acts on the ${\text{M}}_{\text{z}}$ component of each ${\text{M}}_{\text{i}}$ according to:

(2) $$\begin{array}{l} {\left[\begin{array}{c} M_{z,\mathrm{MY}}\\ M_{z,\mathrm{CL}}\\ M_{z,\mathrm{FW}}\\ M_{z,\mathrm{EPW}} \end{array}\right]}_{i,n+1}=\\ \left[\begin{array}{cccc} E_{1,\mathrm{MY}}\;-\;S_{\mathrm{MY}}\left(1\;-\;K_{\mathrm{MC}}\right) & S_{\mathrm{MY}}\left(1\;-\;K_{\mathrm{MC}}\right) & 0 & 0\\ S_{\mathrm{CLa}}\left(1\;-\;K_{\mathrm{MC}}\right) & E_{1,\mathrm{CL}}\;-\;S_{\mathrm{CLa}}\left(1\;-\;K_{\mathrm{MC}}\right)\;-\;S_{\mathrm{CLb}} & 0 & S_{\mathrm{CLb}}\\ 0 & 0 & E_{1,\mathrm{FW}} & 0\\ 0 & S_{\mathrm{EPW}} & 0 & E_{1,\mathrm{EPW}}\;-\;S_{\mathrm{EPW}} \end{array}\right]*{\left[\begin{array}{c} M_{z,\mathrm{MY}}\\ M_{z,\mathrm{CL}}\\ M_{z,\mathrm{FW}}\\ M_{z,\mathrm{EPW}} \end{array}\right]}_{i,n}+\left[\begin{array}{c} 1\;-\;E_{1,\mathrm{MY}}\\ 1\;-\;E_{1,\mathrm{CL}}\\ 1\;-\;E_{1,\mathrm{FW}}\\ 1\;-\;E_{1,\mathrm{EPW}} \end{array}\right] \end{array}$$

where ${\text{E}}_{\text{1,MY}}$ = exp(−tR_1,MY_), ${\text{E}}_{\text{1,CL}}$ = exp(−tR_1,CL_), ${\text{E}}_{\text{1,FW}}$ = exp(−tR_1,FW_), ${\text{E}}_{\text{1,EPW}}$ = exp(−tR_1,EPW_) and ${\text{K}}_{\text{MC}}$ = exp(−t*k*_MY-CL_). The exchange rate ${\text{K}}_{\text{MC}}$ is the combined forward and backward exchange rate between V_MY_ and V_CL_. The exchange rate between V_EPW_ and V_CL_ is infinitely high. The scaling factors *S*_MY_ = V_CL_ * PD_CL_/(V_MY_ * PD_MY_ + V_CL_ * PD_CL_), ${\text{S}}_{\text{CLa}}$ = V_MY_ * PD_MY_/ (V_MY_ * PD_MY_ + V_CL_ * PD_CL_), ${\text{S}}_{\text{CLb}}$ = V_EPW_ * PD_EPW_/(V_EPW_ * PD_EPW_ + V_CL_ * PD_CL_) and *S*_EPW_ = V_CL_ * PD_CL_/(V_EPW_ * PD_EPW_ + V_CL_ * PD_CL_) are required to take the relative volumes of PD in each compartment into account.

***R***_R2_ is the relaxation matrix for the elements for the transverse relaxation rate R_2_. ***R***_R2_ only acts on the ${\text{M}}_{\text{xy}}$ component of each ${\text{M}}_{\text{i}}$ according to:

(3) $${\left[\begin{array}{c} M_{\mathrm{xy},\mathrm{MY}}\\ M_{\mathrm{xy},\mathrm{CL}}\\ M_{\mathrm{xy},\mathrm{FW}}\\ M_{\mathrm{xy},\mathrm{EPW}} \end{array}\right]}_{i,n+1}=\left[\begin{array}{cccc} E_{2,\mathrm{MY}}-S_{\mathrm{MY}}\left(1-K_{\mathrm{MC}}\right) & S_{\mathrm{MY}}\left(1-K_{\mathrm{MC}}\right) & 0 & 0\\ S_{\mathrm{CLa}}\left(1-K_{\mathrm{MC}}\right) & E_{2,\mathrm{CL}}-S_{\mathrm{CLa}}\left(1-K_{\mathrm{MC}}\right)-S_{\mathrm{CLb}} & 0 & S_{\mathrm{CLb}}\\ 0 & 0 & E_{2,\mathrm{FW}} & 0\\ 0 & S_{\mathrm{EPW}} & 0 & E_{2,\mathrm{EPW}}-S_{\mathrm{EPW}} \end{array}\right]*{\left[\begin{array}{c} M_{\mathrm{xy},\mathrm{MY}}\\ M_{\mathrm{xy},\mathrm{CL}}\\ M_{\mathrm{xy},\mathrm{FW}}\\ M_{\mathrm{xy},\mathrm{EPW}} \end{array}\right]}_{i,n}$$

where ${\text{E}}_{\text{2,MY}}$ = exp(−tR_2,MY_), ${\text{E}}_{\text{2,Cl}}$ = exp(−tR_2,CL_), ${\text{E}}_{\text{2,FW}}$ = exp(−tR_2,FW_), ${\text{E}}_{\text{2,ECB}}$ = exp(−${\text{tR}}_{\text{2,ECB}}$).

### MR Quantification Sequence

The presented Bloch equations form a general description of the magnetization evolution for each acquisition voxel and only have meaning when applied to an actual MRI sequence. The specifics of this MRI sequence, with the applied RF pulses, gradients, and timings, dictate the observable signal behavior. The MRI quantification method employed was a multi-echo, multi-delay saturation recovery spin echo sequence (QRAPMASTER) as described previously (20). It was a multi-slice sequence where slice-selective saturation pulses were interleaved with a CPMG acquisition of 5 echoes at 14-ms multiples. The saturation pulse acted on slice *n*, whereas the subsequent acquisition acted on slice *m*. By a fixed shift between slices *n* and *m*, an effective delay time TD was created between the saturation and acquisition of each particular slice. The sequence was repeated four times where the shift between *n* and *m*, and hence the saturation delay, was changed. The result of the sequence was a matrix of 20 images at five different echo times TE and at four different saturation delay times TD. The applied slice-selective RF pulse profiles and amplitudes, gradient strengths, and timings were extracted from the scanner. The repetition time TR was 2950 ms with 30 slices of 4-mm thickness with an in-plane resolution of 1 mm. The saturation pulse had a flip angle of 120° over the *x*-axis followed by a delay of 100, 400, 1380, and 2860 ms, corresponding to a shift between *n* and *m* of 1, 4, 14, and 29 slices, respectively. The excitation pulse had a flip angle of 90° over the x-axis, followed by refocusing pulses of 180° over the y-axis. The refocusing pulses were straddled by spoiler gradients. The scan time was 8:21 min on a Philips Achieva 1.5T (Philips Healthcare, Best, The Netherlands).

### Application of the Bloch Simulation on the Quantification Sequence

The RF pulses, gradients, and timings of the quantification sequence were implemented as a script into the model calculations. The product of all matrices in Eq. 1 does not commute (AB ≠ BA) and, therefore, Eq. 1 is only valid if time-steps are chosen such that the relaxation rates cause a near-zero change of magnetization per time step. Typical relaxation in the brain occurs in the order of millisecond. Therefore, we choose time steps *t* of 1 μs, which is three orders of magnitude smaller, but still results in a reasonable calculation time. The observable signal intensity *I* at each combination of TE and TD was calculated as the product of the total proton density for each partial volume (V * PD) and the *xy*-component of the magnetization ${\text{M}}_{\text{i}}$ of these spins, summed over all elements i:

(4) $$\begin{array}{llll} I_{\mathrm{TE},\mathrm{TD}} & =\sum_{i}\;(V_{\mathrm{MY}}*PD_{\mathrm{MY}}*M_{\mathrm{xy},\mathrm{MY}}+V_{\mathrm{CL}}*PD_{\mathrm{CL}}*M_{\mathrm{xy},\mathrm{CL}}\; & & \;\\ & \;+V_{\mathrm{FW}}*PD_{\mathrm{FW}}*M_{\mathrm{xy},\mathrm{FW}}\; & & \;\\ & \;\;+\;V_{\mathrm{EPW}}*PD_{\mathrm{EPW}}*M_{\mathrm{xy},\mathrm{EPW}})\mathrm{TE},\mathrm{TD} \end{array}$$

In this way, the Block simulation also produced 20 images with different TE and TD, identical to the *in vivo* quantification sequence.

### Subjects

MR quantification was performed on two groups of subjects, one with 20 patients diagnosed with Clinically Definite Multiple Sclerosis (5 males and 15 females; mean age of 47 ± 12 years). The mean extended disability status scale [EDSS (24)] of the MS group was 3.6 ± 2.2, and the mean disease duration was 15 ± 10 years. The second group consisted of age- and gender-matched healthy controls (5 males and 15 females; mean age of 47 ± 11 years). Three female participants were used as individual examples: one healthy subject of 45 years old, one healthy subject of 72 years old, and a secondary progressive MS patient of 45 years old (EDSS of 3.5; disease duration of 17 years). The study was approved by the regional ethical review board and written informed consent was obtained from all participants (full name of the board: “Regionala etikprövningsnämnden i Linköping”; registered under number Dnr. M88-07).

### Image Post-Processing

R_1_, R_2_, and PD maps were retrieved from both the simulated and *in vivo* acquired images using SyMRI 7.0 (SyntheticMR, Linköping, Sweden). In summary, a least squares fit was performed as a function of the different TE and TD times according to:

(5) $$\begin{array}{ll} & I_{\mathrm{TE},\mathrm{TD}}=A.\mathrm{PD}.\exp\left(-R_{2}\mathrm{TE}\right)\times \frac{1-\left[1-\cos\left(B_{1}\theta \right)\right].\exp\left(-R_{1}\mathrm{TD}\right)-\cos\left(B_{1}\theta \right).\exp\left(-R_{1}\mathrm{TR}\right)}{1-\cos\left(B_{1}\alpha \right).\cos\left(B_{1}\theta \right).\exp\left(-R_{1}\mathrm{TR}\right)} \end{array}$$

where α is the excitation flip angle, θ is the saturation flip angle, and B_1_ is the amplitude of the B_1_ field. A is an overall scaling factor that considers the coil sensitivity, RF chain amplification, and voxel volume (20). This equation explicitly has two mono-exponential functions, in R_1_ and R_2_, and hence it will reflect the dominant component of the relaxation behavior.

For spatial normalization of the *in vivo* brain data, the R_1_, R_2_, and PD maps were used to synthesize a stack of T_2_-weighted images with TE = 100 ms and TR = 4500 ms. The synthetic T_2_-weighted images were smoothed with an 8-mm Gaussian kernel and used as source images to calculate the transformation matrix to a standard stereotactic space in Montreal Neurological Institute (MNI) coordinates (26). The images were then transformed to match the size and position of a standard template using a 12-parameter (translation, rotation, shear, zoom) affine regularization and non-linear deformations by a linear combination of three-dimensional discrete cosine basis functions. The same transformation matrix was then applied to the R_1_, R_2_, and PD maps. The resulting data were re-gridded to 2 mm × 2 mm × 2 mm to obtain an isotropic dataset. All of the subjects were averaged to obtain the mean R_1_–R_2_–PD values of the MS and control group. Finally, the mean R_1_, R_2_, and PD values were used as coordinates in a R_1_–R_2_–PD multi-parametric space, as presented in Ref. (21). The 2D histograms of the entire brain were created with 200 bins for R_1_ on a scale of 0–2 s^−1^, 200 bins for R_2_ on a scale of 0–15 s^−1^, and 200 bins for PD on a scale of 50–100%.

### Determining the Model Parameters

**Figure 2:**
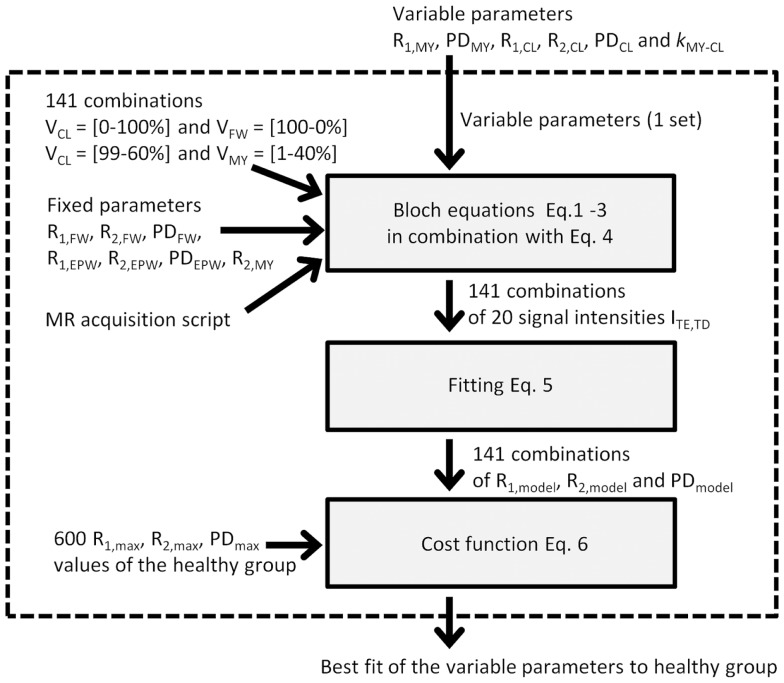
**Schematic depiction of the procedure to optimize the variable parameters: One set of variable parameters is chosen and evaluated within the dotted box**. Evaluation is performed by running the Bloch equations of the simulated MR acquisition on 141 combinations of V_MY_, V_CL_, and V_FW_. This provides 20 signal intensities at various echo times and saturation delays times. The 20 signal intensities are fitted, resulting in an R_1_, R_2_, and PD value of the model. The model values are then compared to the observed R_1_, R_2_, and PD values of the healthy group using the maximum values in the 2D histograms. A cost function provides a measure for closeness of the model R_1_, R_2_, and PD values to the observed R_1_, R_2_, and PD values. The evaluation is performed for many sets of variable parameters, resulting in the best fit.

The procedure to determine the model parameters is schematically depicted in Figure 2. In the model, the relaxation parameters for water, both for V_FW_ and V_EPW_, were fixed to literature values for CSF at R_1_ = 0.24 s^−1^, R_2_ = 0.87 s^−1^, and PD = 100% (20). Additionally, the R_2_ relaxation for V_MY_ was fixed to a reported value, at R_2,MY_ = 77 s^−1^ (corresponding to T_2,MY_ = 13 ms) (22). Therefore, only six remaining model parameters, R_1,MY_, PD_MY_, R_1,CL_, R_2,CL_, PD_CL_, and *k*_MY-CL_, were allowed to vary. The six model parameters were given a random value under the restriction that R_1,FW_ < R_1,CL_ < R_1,MY_ and R_2,FW_ < R_2,CL_ < R_2,MY_. For each set of variable parameters, the magnetization evolution was calculated for all combinations of V_MY_ and V_CL_ and for all combinations of V_CL_ and V_FW_, using steps of 1% partial volume. Since the maximum amount is 100%, a setting of for example 20% V_FW_ requires a setting of 80% V_CL_, hence producing 101 combinations of V_FW_ and V_CL_. V_MY_ was restricted to a maximum of 40%, since no higher values were expected to occur in the brain and we wanted to avoid values that could not be evaluated. This produced 40 combinations of V_MY_ and V_CL_, making a total of 141 combinations. The magnetization evolution was calculated using Eqs 1–3, resulting in the signal intensities ${\text{I}}_{\text{TE,TD}}$ at five different echo times TE and four different saturation delay times TD for each partial volume combination (Eq. 4). The sets of 20 ${\text{I}}_{\text{TE,TD}}$ values were then fitted using Eq. 5, resulting in 141 ${\text{R}}_{\text{1,model}}$, ${\text{R}}_{\text{2,model}}$, and ${\text{PD}}_{\text{model}}$ values for each specific set of variable parameters.

To evaluate how close these 141 R_1_–R_2_–PD values mimicked the observed data structure in the 2D histograms of the healthy control group, the maximum values in the histogram for each bin in R_1_ were determined, and the corresponding R_2_ and PD values were recorded. This procedure was repeated for R_2_ and PD. Because the 2D histograms had 200 × 200 bins, this procedure provided 600 ${\text{R}}_{\text{1,max}}$, ${\text{R}}_{\text{2,max}}$, and ${\text{PD}}_{\text{max}}$ values to define the characteristic data structure of the healthy group. From these 600 combinations, 141 were selected that were closest to the 141 model combinations.

Finally, a cost function was set up to evaluate the difference between the ${\text{R}}_{\text{1,model}}$, ${\text{R}}_{\text{2,model}}$, and ${\text{PD}}_{\text{model}}$ values for each parameter setting with the selected ${\text{R}}_{\text{1,max}}$, ${\text{R}}_{\text{2,max}}$, and ${\text{PD}}_{\text{max}}$ values of the 2D histograms of the *in vivo* spatially normalized data:

(6) $$\begin{array}{llll} f_{\mathrm{cost}} & \;=\;\frac{1}{n}\sum {\left[{\left(\frac{R_{2,\mathrm{model}}-R_{2,\max}}{\sigma \left(R_{2}\right)}\right)}^{2}+{\left(\frac{PD_{\mathrm{model}}-PD_{\max}}{\sigma \left(\mathrm{PD}\right)}\right)}^{2}\right]}_{R_{1}}\; & & \;\\ & \;+{\left[{\left(\frac{R_{1,\mathrm{model}}-R_{1,\max}}{\sigma \left(R_{1}\right)}\right)}^{2}+{\left(\frac{PD_{\mathrm{model}}-PD_{\max}}{\sigma \left(\mathrm{PD}\right)}\right)}^{2}\right]}_{R_{2}}\; & & \;\\ & \;+{\left[{\left(\frac{R_{1,\mathrm{model}}-R_{1,\max}}{\sigma \left(R_{1}\right)}\right)}^{2}+{\left(\frac{R_{2,\mathrm{model}}-R_{2,\max}}{\sigma \left(R_{2}\right)}\right)}^{2}\right]}_{\mathrm{PD}} \end{array}$$

To ensure that R_1_, R_2_, and PD had the same weight in the cost function, the square of the residuals was normalized using the variance σ^2^ of R_1_, R_2_, and PD (27).

The entire procedure was repeated, where each of the variable parameters was varied individually, with increasingly smaller steps until the minimum residual was found. To avoid convergence to a local minimum, this procedure was repeated 100 times, after which the lowest residual was regarded as the global minimum.

The confidence interval of the optimized parameters was calculated using the finite sample confidence intervals in the maximum likelihood (25). According to this approach, the confidence region is found by varying a single parameter and minimizing all others such that the cost function remains under the value of χ^2^(*a, df*), where *a* corresponds to the confidence level and *df* is the number of degrees of freedom. Using *a* = 0.05 and *df*  = 5, the χ^2^(*a, df*) function becomes 9.488. The Bloch simulation and minimization procedure was implemented in an in-house developed IDL program (ITT visual information solutions, Boulder, CO, USA).

### Calculation of Total Volumes and Regions of Interest

Segmentation of the intracranial volume (ICV) was performed using an automatic procedure in SyMRI 7.0. The total myelin volume (MYV), cellular volume (CV), free water volume (FWV), and excess parenchymal water volume (EPWV) were calculated by summing all partial volumes within the ICV. The brain parenchymal volume (BPV) was defined as the ICV minus the total FWV. The brain parenchymal fraction (BPF) corresponds to BPV divided by ICV. The myelin fraction (MYF) was calculated as the total MYV divided by the BPV. Also, the cellular water fraction (CF) and excess parenchymal water fraction (EPWF) were calculated in a similar manner as the total CV divided by the BPV and total EPWV divided by the BPV, respectively.

The MWF can be derived from the model parameters because the MyW corresponds to the PD_MY_ in the V_MY_, and the intra- and extracellular water corresponds to the sum of PD_CL_ and PD_EPW_ in the V_CL_ and V_EPW_, such that MWF for each acquisition voxel can be calculated as MWF = (V_MY_ * PD_MY_)/(V_CL_ * PD_CL_ + V_EPW_ * PD_EPW_). Additionally, the total aqueous content of the tissue can be calculated, corresponding to the sum of the MyW, cellular water, free water, and excess parenchymal water, V_MY_ * PD_MY_ + V_CL_ * PD_CL_ + V_FW_ * PD_FW_ + V_EPW_ * PD_EPW_. The total non-aqueous content then corresponds to 100% minus the aqueous content.

To define regions of interest for the spatially normalized brain images, the cropped ROI templates, based on the Wake Forrest University (WFU) PickAtlas, were taken [Ref. (21)]. To verify that the standard ROIs in spatially normalized, averaged brain images provide similar results as spatially non-normalized, separate brain images, 3 mm × 3 mm ROIs were manually placed in a subset of brain structures in all participants of Ref. (21). This was also done for the three example subjects. In the MS cases, areas with MS lesions were avoided.

## Results

### Optimizing the Model Parameters to the Healthy Brain

**Figure 3:**
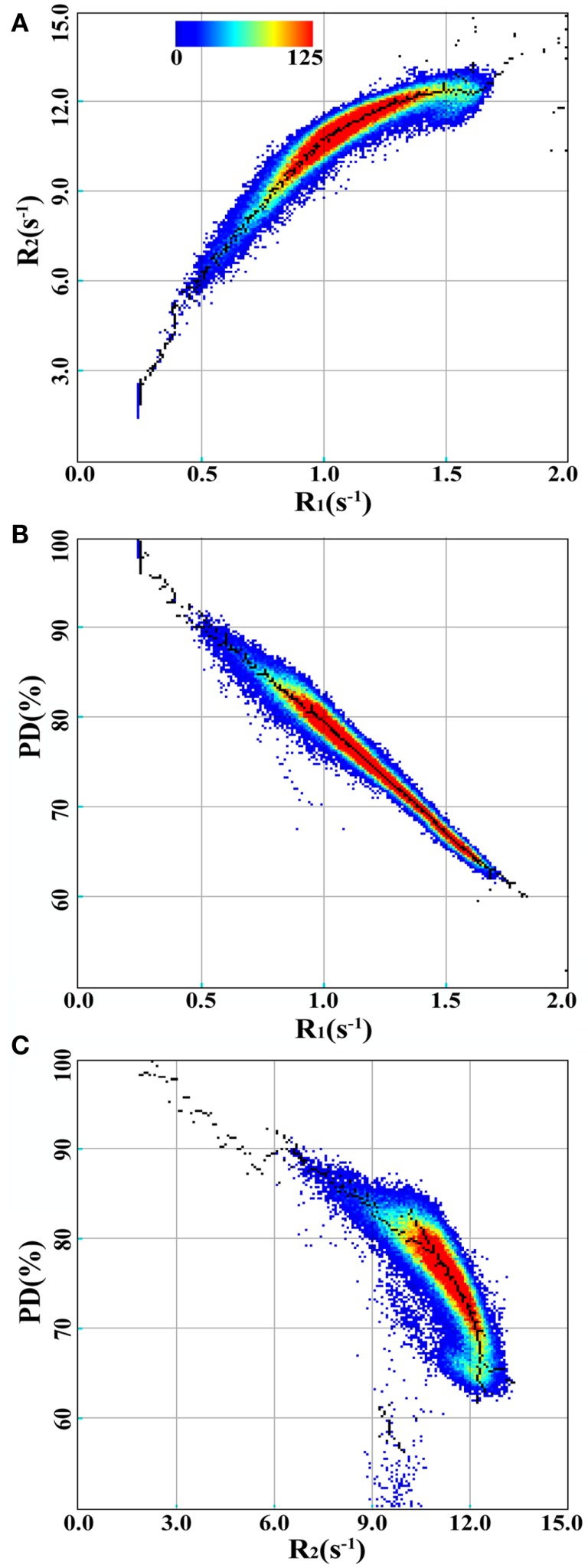
**2D-histograms of R_1_, R_2_, and PD values for the spatially normalized brain images of the group of control subjects**. The 2D-histograms of R_1_ and R_2_, R_1_ and PD, and R_2_ and PD are shown in **(A–C)**, respectively. The color scale indicates the number of voxels for each coordinate. The black dots are placed at the maximum values of the 2D-histograms in each direction.

In Figure 3, the R_1_, R_2_, and PD values for the spatially normalized brains of the group of controls are shown as 2D histograms of R_1_ and R_2_, R_1_ and PD, and R_2_ and PD. The color scale indicates the number of voxels for each coordinate in the histogram. The black dots are placed at the maximum values of the histograms in each direction, generating the 600 maxima defining the structure in the R_1_–R_2_–PD space.

Using these 600 maxima, the six variables in the model were optimized to find the minimum value of the cost function (See Figure 2). The values of the parameters at the minimum residual (3.446) are given in Table 1. Each parameter was varied individually while re-optimizing all others such that the cost function remained below 9.488, resulting in the determination of the SDs of the parameters, as also listed in Table 1.

**Table 1:** **The parameter values of the model; on the left the fixed parameters (see MATERIALS AND METHODS); on the right, the optimized parameters where the cost function was minimized for the brain data of the control group (*n* = 20)**.

Fixed parameters	Optimized parameters
R_2,MY_ = 77 s^−1^	R_1,MY_ = 16.6 ± 13.2 s^−1^
R_1,FW_ = R_1,EPW_ = 0.24 s^−1^	PD_MY_ = 42 ± 33%
R_2,FW_ = R_2,EPW_ = 0.87 s^−1^	*k*_VMY-VCL_ = 6.7 ± 5.2 s^−1^
PD_FW_ = PD_EPW_ = 100%	R_1,CL_ = 0.78 ± 0.13 s^−1^
*k*_VEPW-VCL_ = ∞s^−1^	R_2,CL_ = 10.3 ± 0.6 s^−1^
	PD_CL_ = 85 ± 5%

*The SD of the latter values is given for a significance level of *a* = 0.05*.

### Behavior of the Model for the Pathological Brain

**Figure 4:**
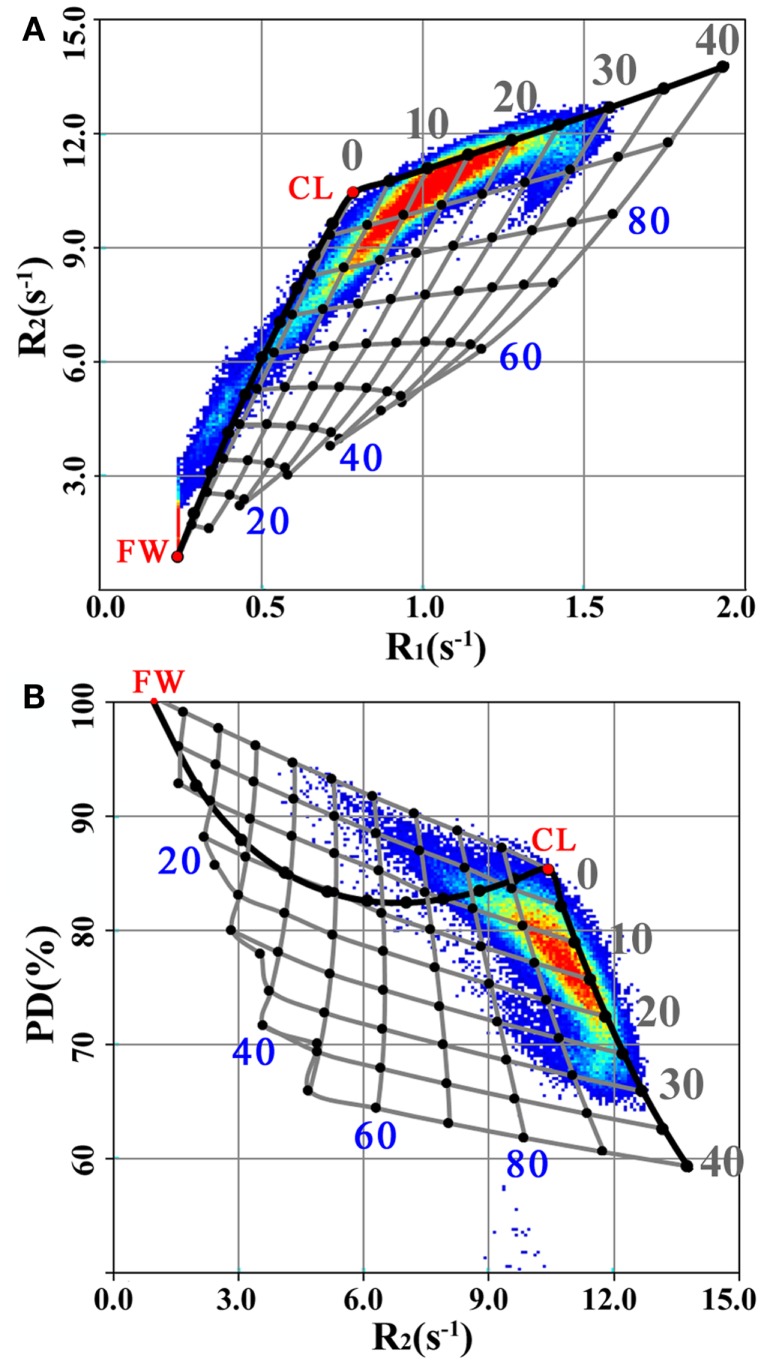
**R_1_, R_2_, and PD values for the spatially normalized brain images of the group of MS patients, plotted in the same manner as Figure 3 for the R_1_–R_2_ (A) and R_2_–PD (B) projections**. Additionally, the thick black line indicates the transition from 100% V_FW_ (the red dot at “FW”) to 100% V_CL_ (the red dot at “CL”) until 40% V_MY_, using the model parameter settings for the healthy controls (Table 1). The grid of gray lines indicates the expected changes in R_1_, R_2_, and PD values for the pathological brain under myelin loss (Figure 1C) and under the presence of excess parenchymal water (Figure 1D). The cross points of the grid are placed at each 5% change in V_MY_ and each 10% change in V_EPW_. The V_MY_ partial volume is indicated by the gray numbers 0–40%. The V_EPW_ partial volume is indicated by the blue numbers 20–80%.

The mean values in Table 1 provide the relaxation parameters for the four partial volumes for the healthy brain. According to the model, all observed R_1_, R_2_, and PD values in the healthy brain can be reproduced by combinations of V_FW_, V_CL_, and V_MY_ using these characteristics. This is indicated as the thick black curve in Figure 4 showing the transition from 100% V_FW_ at (R_1_, R_2_, PD) = (0.24 s^−1^, 0.87 s^−1^, 100%) to 100% V_CL_ at (R_1_, R_2_, PD) = (0.78 s^−1^, 10.3 s^−1^, 85%), continuing toward 100% V_MY_ at (R_1_, R_2_, PD) = (16.6 s^−1^, 77 s^−1^, 42%). In the Figure, the positions of 100% V_FW_ and 100% V_CL_ are indicated at the red dots labeled by “FW” and “CL,” respectively. The 100% V_MY_ position is outside the range of the plot, the grid is clipped at 40% V_MY_.

For the pathological brain, two processes can occur in the model: (1) a decrease in V_MY_ and (2) the presence of non-zero V_EPW_. In Figure 4 a grid is displayed, indicating steps of possible combinations of 5% difference of V_MY_ and 10% difference of V_EPW_. This grid spans a curved surface in the R_1_–R_2_–PD space. In the background of Figure 4 the data for the spatially normalized brain for the MS group were plotted. It can be seen that the MS data values are shifted toward lower R_1_ and R_2_ and higher PD relative to the black curve, which was optimized using the healthy data values.

### Modeling the Spatially Normalized Brain Images

**Figure 5:**
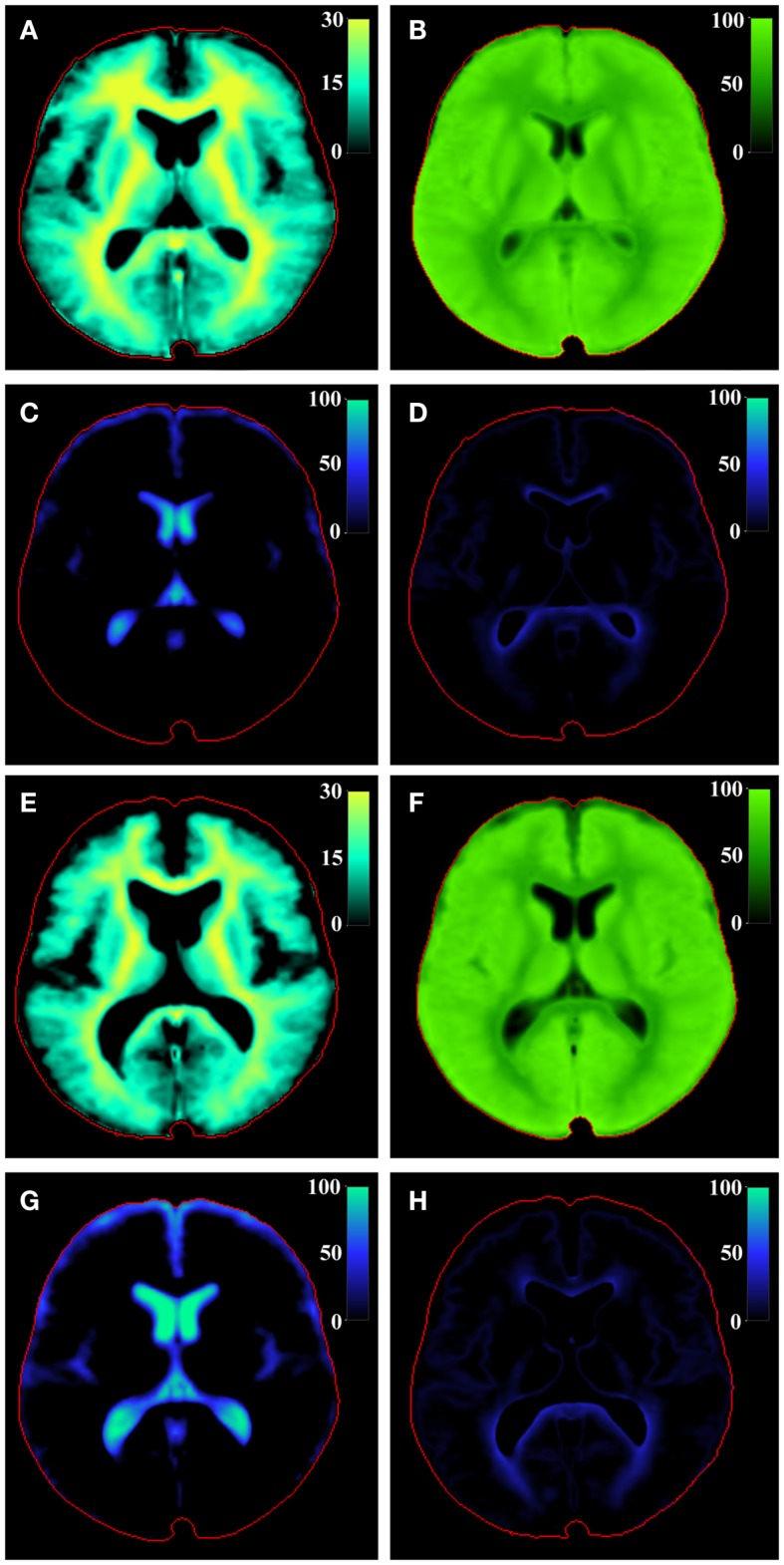
**Model calculation of (A) V_MY_, (B) V_CL_, (C) V_FW_, and (D) V_EPW_ of the central slice of the brain of the spatially normalized group of healthy controls and of the spatially normalized group of MS patients [(E–H), respectively]**. The red line indicates the intracranial volume. Note that V_MY_ is scaled to 30%, whereas the other partial volumes are scaled to 100%.

The grid in Figure 4 was used to relate the R_1_, R_2_, and PD values of the spatially normalized brain data to combinations of V_MY_, V_CL_, V_FW_, and V_EPW_. The result is shown in Figure 5 for the spatially normalized brain images of the control and MS groups. The V_MY_ is substantially higher for the controls than for the MS group. The total MYVs were 157 and 119 mL, respectively, a difference of 38 mL. Also, the total FWV was visibly lower, at 65 mL for the control group versus 144 mL for the MS group, a difference of 79 mL. The ICV of the spatially normalized datasets was 1213 mL for both groups, resulting in brain volumes of 1148 and 1069 mL, corresponding to a BPF of 94.6 and 88.1%, respectively. All volumes and volume fractions in relation to brain volume are provided in Table 2.

**Table 2:** **The total volumes and volume fractions for the spatially normalized healthy control group and spatially normalized MS group of Figures 4 and 5 as well as for the three individual subjects of Figure 6**.

	MYV (mL)	CV (mL)	FWV (mL)	EPWV (mL)	BPV (mL)	ICV (mL)	MYF (%)	CF (%)	EPWF (%)
Control	157	934	65	57	1148	1213	13.7	81.4	5.0
MS	119	872	144	78	1069	1213	11.1	81.6	7.3
45 year	155	911	117	24	1090	1207	14.2	83.6	2.2
72 year	142	944	308	41	1127	1435	12.6	83.7	3.7
45 year-MS	119	875	204	37	1031	1234	11.5	84.9	3.6

*Listed are the total myelin volume (MYV), cellular volume (CV), free water volume (FWV), excess parenchymal water volume (EPWV), total brain volume (BPV), and intracranial volume (ICV). The volume components that constitute the brain were normalized on BPV, resulting in the myelin fraction (MYF), cellular fraction (CF), and excess parenchymal water fraction (EPWF) of the brain*.

The observed R_1_, R_2_, and PD values in the standard WFU PickAtlas ROIs of separate brain structures were used to calculate the local mean V_MY_, V_CL_, and V_EPW_ of the spatially normalized control group and spatially normalized MS group (see Table 3). For the healthy group, V_MY_ for the GM structures was in the range of 8–15% (average 14 ± 3%), whereas that for WM structures was 18–27% (average 23 ± 3%). For the MS group, V_MY_ was 1–4% lower, with most of the difference in the WM structures; the average was 13 ± 5% for GM structures (difference: 1.6 ± 1.5%) and 20 ± 3% for WM structures (difference: 2.8 ± 1.0%). The mean V_CL_ was 0–10% lower in the MS group. V_EPW_ was higher in the MS group, with a difference of 9 ± 10% and 5 ± 2%, respectively, compared to the healthy group. Large differences were observed for the caudate nucleus, for which the MS group had a 28% lower V_CL_ and 31% higher V_EPW_ compared with the healthy group. For completeness, also the MWF was derived from the model, which was 8.3 ± 2.9% for GM structures and 14.4 ± 2.3% for WM structures for the healthy group and 7.2 ± 3.0% and 11.9 ± 2.3%, respectively, for the MS group, a difference of 1.2 ± 0.9% and 2.5 ± 0.7%, respectively. The MWF values show the same trend as V_MY_ but are substantially lower, 43% on average.

**Table 3:** **The mean myelin partial volume V_MY_, cellular partial volume V_CL_, and the excess parenchymal water partial volume V_EPW_ of various brain structures, estimated as a percentage of the acquisition voxel volume**.

	Healthy controls				Multiple sclerosis patients			
	
	
	V_MY_ (%)	V_CL_ (%)	V_EPW_ (%)	MWF (%)	V_MY_ (%)	V_CL_ (%)	V_EPW_ (%)	MWF (%)
Insula	8	75	17	4	8	66	26	4
Cingulate cortex	12	81	7	7	8	78	14	4
Caudate nucleus	9	87	4	5	6	59	35	3
Cortical gray matter	15	74	11	9	14	66	20	8
Pons	18	69	13	11	17	60	23	10
Putamen	15	85	0	9	15	85	0	9
Mid brain	19	81	0	12	18	79	3	11
Thalamus	19	81	0	12	16	84	0	9
Occipital white matter	18	82	0	11	15	83	2	9
Frontal white matter	21	77	2	14	19	73	8	11
Parietal white matter	21	77	2	14	19	73	8	11
Sub-lobar white matter	25	66	9	16	21	65	14	13
White matter	23	75	2	15	19	73	8	11
Corpus callosum	27	60	13	18	25	55	20	16

*The values were calculated using the proposed model and the reported relaxation rates R_1_ and R_2_ and proton density PD in the WFU Pickatlas ROIs of the spatially normalized, averaged group of healthy controls and the spatially normalized, averaged group of multiple sclerosis patients from Ref. (21) (Table 2, cropped ROI templates). Added are the expected myelin water fraction MWF values, calculated as PD_MY_/(PD_CL_ + PD_EPW_)*.

For comparison, ROIs were manually placed in a subset of all brain structures for all participants in the study, using the original, spatially non-normalized brain images (Table 4). The differences between GM and WM structures are far more extreme in this case. For example, for the healthy group, the V_MY_ for cortical GM decreases from 15% for the standard ROI to 2% for the manually placed ROI, whereas for the corpus callosum V_MY_ increases from 27 to 41%. Most of the V_EPW_ values decrease, except for the occipital WM (9%). For the manual ROIs, no significant differences were observed for the GM structures between the MS patients and the control group. For WM, however, V_MY_ was 3% lower for occipital WM (*p* = 0.04), 2% lower for frontal WM (*p* = 0.04), and 5% lower for corpus callosum (*p* = 0.02).

**Table 4:** **The mean myelin partial volume V_MY_, cellular partial volume V_CL_, and the excess parenchymal water partial volume V_EPW_ of various brain structures, estimated as a percentage of the acquisition voxel volume**.

	Healthy controls				Multiple sclerosis patients			
	
	
	V_MY_ (%)	V_CL_ (%)	V_EPW_ (%)	MWF (%)	V_MY_ (%)	V_CL_ (%)	V_EPW_ (%)	MWF (%)
Cingulate cortex	2	96	2	1	2	95	3	1
Caudate nucleus	8	92	0	4	9	91	0	5
Cortical gray matter	2	95	3	1	2	95	3	1
Putamen	11	89	0	6	10	90	0	5
Thalamus	19	81	0	12	15	84	1	9
Occipital white matter	34	57	9	25	31	61	8	22
Frontal white matter	36	62	2	28	34	64	2	25
Corpus callosum	41	56	3	35	36	60	4	29

*The values were calculated using the proposed model and the relaxation rates R_1_ and R_2_ and proton density PD in manually placed ROIs in all participants of Ref. (21). Added are the expected myelin water fraction MWF values, calculated as PD_MY_/(PD_CL_ + PD_EPW_)*.

### Modeling the High-Resolution Brain Images

**Figure 6:**
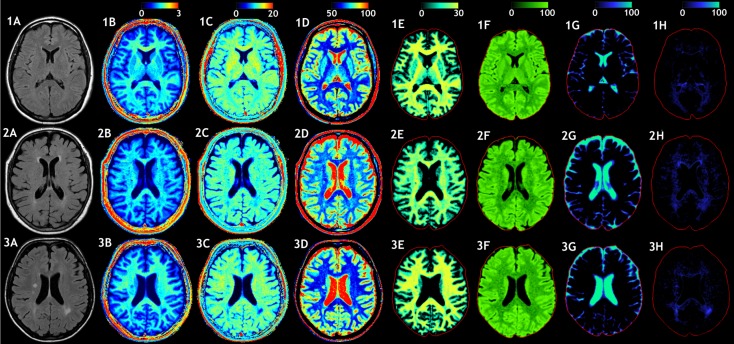
**Examples of the model calculation on an axial slice of the brain**. (row 1) Healthy subject, female 45 years old, (row 2) elderly control subject, female 72 years old, and (row 3) patient, female, 45 years old, diagnosed with secondary progressive MS. **(A)** A conventional FLAIR image of the same slice is added as a visual reference. **(B)** The measured R_1_ relaxation rate is shown on a scale of 0–3 s^−1^, **(C)** the R_2_ relaxation rate is shown on a scale of 0–20 s^−1^, and **(D)** the proton density PD is shown on a scale of 50–100%, where 100% corresponds to pure water at 37°C. **(E)** Using the R_1_, R_2_, and PD values in combination with the look-up grid of Figure 4 the myelin partial volume V_MY_ was calculated, as shown on a scale of 0–30%, **(F)** the cellular partial volume V_CL_, **(G)** free water partial volume V_FW_, and **(H)** excess parenchymal water partial volume V_EPW_ were all calculated all on a scale 0–100%. The red intracranial cavity outline is displayed in all tissue images for visual guidance.

In Figure 6, the model was applied on high-resolution image datasets of a middle-aged (45 years) and elderly control subject (72 years) and an MS patient (45 year-MS), in combination with a conventional FLAIR image (A). The R_1_, R_2_, and PD maps (B–D) demonstrate that the 72 year (row 2) had generally lower R_1_ and R_2_ values and higher PD values throughout the brain than the 45 year (row 1). For the 45 year-MS (row 3), the R_1_, R_2_, and PD values were similar to those for the 45 year, but much lower in the areas where the MS lesions were located. Figure 6E presents the estimated V_MY_, with a high V_MY_ in the WM (33%, see Table 5) and low V_MY_ in the GM (4%) for the 45 year. The 72 year showed less myelin throughout the brain than the 45 year, with an average V_MY_ of 26% in the WM. Only the corpus callosum showed higher values (33%). The estimated total MYVs were 155 mL for the 45 year, 142 mL for the 72 year and 119 mL for the 45 year-MS, corresponding to a MYF of 14.2, 12.6 and 11.5%, respectively (see Table 2). The cellular fractions (Figure 6F) were 83.7, 83.7, and 84.9%, respectively. Figure 6G presents V_FW_, highlighting the ventricular system and periphery of the brain. Using the ICV and FWV of the subjects, the BPV can be calculated, which was 1090 mL for the 45 year, 1127 mL for the 72 year, and 1031 mL for the 45 year-MS. Correspondingly, the BPF was 90.3, 78.5, and 83.5%, respectively.

**Table 5:** **The mean myelin partial volume V_MY_, cellular partial volume V_CL_, the excess parenchymal water partial volume V_EPW_, and myelin water fraction MWF of various brain structures, estimated as a percentage of the acquisition voxel volume for the three example subjects**.

	45 years				72 years				45 year-MS			
	
	
	
	V_MY_ (%)	V_CL_ (%)	V_EPW_ (%)	MWF (%)	V_MY_ (%)	V_CL_ (%)	V_EPW_ (%)	MWF (%)	V_MY_ (%)	V_CL_ (%)	V_EPW_ (%)	MWF (%)
Insula	4	95	1	2	3	91	6	2	7	92	1	4
Cingulate cortex	4	95	1	2	6	91	3	3	2	93	5	1
Caudate nucleus	13	87	0	7	9	91	0	5	10	90	0	5
Cortical gray mater	3	94	3	2	7	91	2	4	4	93	3	2
Pons	23	76	1	15	22	76	2	14	22	78	0	14
Putamen	11	89	0	6	9	91	0	5	12	88	0	7
Mid brain	19	81	0	12	18	79	3	11	21	78	1	13
Thalamus	19	81	0	12	20	79	1	12	21	79	0	13
Occipital white mat	31	58	11	22	27	57	16	18	32	56	12	23
Frontal white mater	35	60	5	26	25	61	14	16	36	62	2	27
Parietal white mater	35	61	4	26	26	70	4	17	35	64	1	27
Sub-lobar white mat	32	63	5	23	21	75	4	13	30	70	0	21
White matter	33	59	8	24	26	72	12	15	32	61	7	24
Corpus callosum	31	63	6	22	33	60	7	24	33	54	13	24

The 45 year exhibited a small amount of V_EPW_ (Figure 6H), mainly around the occipital horns of the lateral ventricles, with a maximum of 11% in the occipital WM. The 72 year had elevated V_EPW_ in the complete periventricular region, with values of up to 16% partial volume. The 45 year-MS showed moderate V_EPW_ values at the periventricular area and 12% in the occipital WM. At the location of MS lesions, however, high V_EPW_ values, up to approximately 50% were observed. The V_EPW_ volumes were 24 mL for the 45 y, 41 mL for the 72 y, and 37 mL for the 45 year-MS, corresponding to an EPWF of 2.2, 3.5, and 3.6%, respectively.

**Figure 7:**
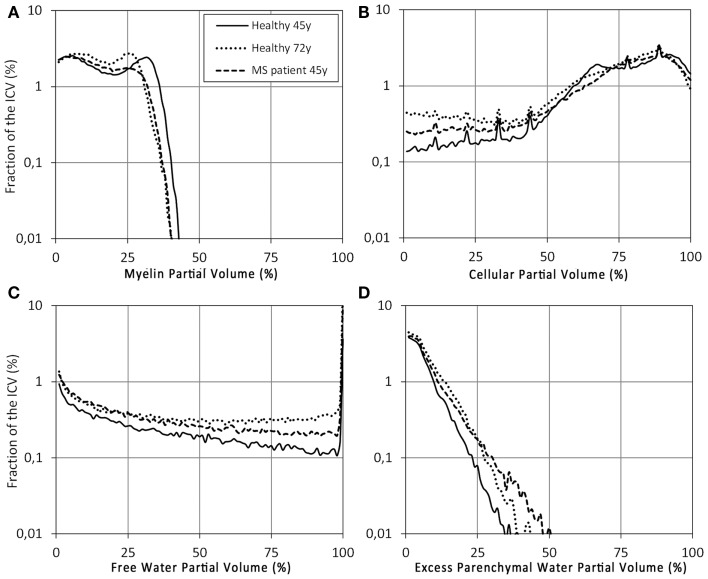
**Histograms of the (A) V_MY_, (B) V_CL_, (C) V_FW_, and (D) V_EPW_ partial volume distributions of the control subject (solid line), elderly control subject (dotted line), and MS patient (dashed line) from Figure 6**. The *x*-axis was divided into 100 bins of 1% partial volume over the range 0–100%. The scaling on the *y*-axis is logarithmic, as a percentage of the ICV.

The histograms of V_MY_, V_CL_, V_FW_, and V_EPW_ are shown in Figure 7 to assess the distribution of the partial volumes of the three subjects. The histograms contain 100 bins from 0 to 100% partial volume and are plotted as a percentage of the ICV volume to compensate for the difference in subject head size. The 45 year exhibited two peaks in the V_MY_ histogram at 5 and 32% V_MY_. For the 72 year, the peak V_MY_ values occurred at 25%. The 45 year-MS did not have a clear peak at higher V_MY_ values. The V_CL_ values peaked at 68 and 92% for the 45 year, but only one peak was observed for both the 72 and 45 year-MS at 89%. V_FW_ values were generally low (<0.5%) in the complete range but exhibited a sharp peak at 100% V_FW_, with a maximum of 3.7% for the 45 year, 23.3% for the 72 year, and 11.9% for the 45 year-MS. V_EPW_ was observed in all three subjects, but the values were lowest for the 45 year.

**Figure 8:**
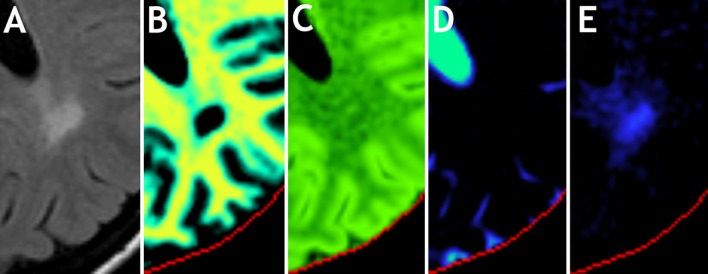
**Zoomed part on an MS lesion of the patient in Figure 6, row 3**. Shown are **(A)** the conventional FLAIR image, **(B)** myelin partial volume V_MY_, **(C)** cellular partial volume V_CL_, **(D)** free water partial volume V_FW_, and **(E)** excess parenchymal water partial volume V_EPW_. Color scaling is identical to Figure 6.

The area with the lesion of the MS patient, posterior to the left lateral ventricle, was zoomed out and displayed in Figure 8, showing a FLAIR image together with V_MY_, V_CL_, V_FW_, and V_EPW_, taken from Figures 6A,E–H. At the location of the FLAIR hyper-intensity, the V_MY_ was equal to 0, whereas the V_EPW_ values were up to 55% partial volume. The diffuse hyper-intensity, located between the lesion and lateral ventricle, exhibited V_MY_ values of 15–20% and V_EPW_ values of 25–30% partial volume. Elevated V_EPW_ values were observed in a large area around the lesion. The V_CL_ varied only slightly, ranging between 45% at the lesion and 55% at the diffusely hyper-intense area.

**Figure 9:**
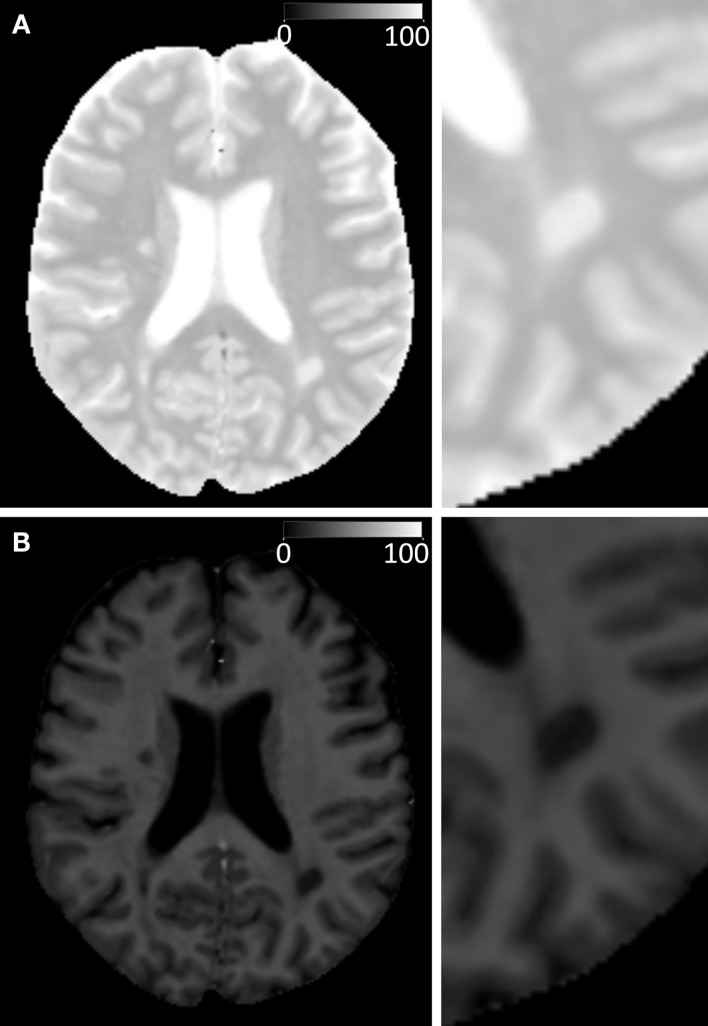
**Calculated total aqueous content (A), corresponding to the sum of myelin water, cellular water, free water and excess parenchymal water, and the remaining, total non-aqueous content (B) of the 45 year-MS patient**. The same slice and zoomed part are displayed as in Figures 6 and 8.

Using the four partial volumes, the total aqueous content of the brain can be derived. The sum of all PD contributions of V_MY_, V_CL_, V_FW_, and V_EPW_ is shown in Figure 9A for the 45 year-MS, for the same slice as Figures 6 and 8. The centers of the MS lesions exhibit a total aqueous content of 85–95%, consisting entirely of the PD component of V_CL_ and V_EPW_. Normal appearing WM for this patient showed not only a total aqueous content approximately 70%, consisting mainly of the PD component of V_MY_ and V_CL_ but also a minor contribution of V_EPW_ in the order of 5%. Normal appearing GM shows a total aqueous content of approximately 85%, consisting largely of the PD component of V_CL_, but with a small contribution of V_MY_, up to 5%. The remaining non-aqueous content is shown in Figure 9B.

## Discussion

In the present study, the R_1_, R_2_, and PD values, as measured in the brain using a fast multi-parametric qMRI sequence, were modeled by four partial volume compartments per acquisition voxel, (1) the myelin partial volume V_MY_, (2) cellular partial volume V_CL_, (3) free water partial volume V_FW_, and (4) excess parenchymal water partial volume V_EPW_. The major advantage of this model is that it produces an estimate of three clinically important parameters, the total brain volume, the degree of myelination of the brain parenchyma, and the degree of edema of the brain parenchyma, based on a single, relatively short acquisition.

For a complex organ, such as the brain, with an abundance of magnetically interacting microscopic substructures, MR signal relaxation will behave as a multitude of exponentials. Multi-component measurements, such as the multi-exponential T_2_ relaxation and mcDESPOT approaches, typically regularize relaxation signals to force the solution into a fast component attributed to MyW, a medium-time component attributed to intra- and extracellular water and occasionally in a long-time component attributed to CSF. Attempts to experimentally resolve the fast component, however, are very challenging. The qMRI sequence employed in this work cannot resolve the fast signal component, but can accurately measure the medium-time relaxation component (28). The estimation of myelin partial volume of our model is, therefore, based on the shift of this medium-time component due to magnetization exchange between MyW and surrounding intra- and extracellular water. Such a shift is observable both in the R_1_ and R_2_ relaxation rates, thus, building a specific pattern in the R_1_–R_2_–PD space, as visualized in Figure 3 for a group of healthy controls and in Figure 4 for a group of MS patients. Therefore, the model relies on a combined R_1_–R_2_–PD measurement as a single component/multi-parametric quantification strategy, in contrast to the multi-component/single parametric quantification methods, such as the multi-component T_2_ relaxation. The observed values for brain parenchyma of R_1_ in the range of 0.9–1.9 s^−1^ (T_1_ = 530–1100 ms) and R_2_ in the range of 10.5–13 s^-1^ (T_2_ = 75–95 ms) corresponded well with previously reported values for GM and WM (29, 30), where other qMRI methods were used. Also, the measured PD corresponds well to the reported values with GM structures of 80–86% and WM of 74–76% (31, 32).

The determined optimal parameter values for the partial volume compartments are listed in Table 1. The result of the optimization provides three specific coordinates in the R_1_–R_2_–PD space, for pure V_FW_ [set by literature values to (R_1_, R_2_, PD) = (0.24 s^−1^, 0.87 s^−1^, 100%)], pure V_CL_ [estimated at (0.78 s^−1^, 10.3 s^−1^, 85%)] and pure V_MY_ [estimated at (16.6 s^−1^, 77 s^−1^, 42%)]. The characteristics of the V_CL_ are close to those of cortical GM (20, 29, 30). The characteristics of the V_MY_ are within the range of previous reported values (11, 22). Using the model, the possible value combinations of R_1_, R_2_, and PD in the healthy brain were visualized by the solid black curve through the R_1_–R_2_–PD space, as plotted in Figure 4. The difference between the healthy brain and pathological brain was described using two components: (1) the variation of the V_MY_, indicating myelin loss, and (2) the presence of V_EPW_, indicating the presence of edema. These two components expanded the (healthy) curve to a curved surface grid, as shown in Figure 4. Each observed value combination of R_1_, R_2_, and PD in acquisition voxels of a pathological brain is regarded as a combination of the V_MY_, V_CL_, V_FW_, and V_EPW_ partial volume compartments. As shown in Figure 5, substantial differences were observed between the spatially normalized control group and spatially normalized MS group in all partial volumes. The MS group had a smaller V_MY_ and V_CL_ (a difference of 3.1 and 5.1% of the ICV, respectively) and larger V_FW_ and V_EPW_ (a difference of 6.5 and 1.7% of the ICV, respectively). Consequently, the average brain volume of the MS group was smaller than that of the control group (88.1% versus 94.6% of the ICV), the degree of myelination in the brain was lower (11.1% versus 13.7% of the BPV) and the degree of edema in the brain was higher (7.3% versus 5.0% of the BPV). This result is congruent with knowledge concerning the disease progression of MS (3–5). The relative CV in the brain was virtually identical (81.6 and 81.4%), as can be expected in a model where edema is described by a separate class of excess parenchymal water, which is an addition of water to the normal cellular partial volume. The values in Table 3 for the various brain structures confirm the image shown in Figure 5.

The model was tested on three individual subjects as examples for high-resolution imaging. This can by no means be representative for entire groups of subjects and, hence, is purely used as example of the application of the model. Inclusion of larger groups to assess statistical differences with different age groups and diseases will be performed in future work. Clear differences were observed among the three subjects. Compared with the healthy controls, the V_MY_ partial volume was lower for both the elderly subject and MS patient (Figure 6). Additionally, the MS patient showed strong local decreases at the location of MS lesions. Similar to the spatially normalized brains of Figure 5, the cellular fraction of the brain was virtually identical for all subjects. The V_FW_ clearly highlights the CSF in the ventricular system and brain periphery, making it possible to calculate the brain volume of the subjects. The elderly subject had the smallest brain, with a BPF of 78.5%, compared with the 90.3% for the healthy 45 year and 83.5% for the MS patient. Simultaneously, the MS patient had the lowest myelination, with a MYF of 11.5%, compared with 14.2% for the healthy 45 year and 12.6% for the 72 year. In Figure 7 the cause of the reduction can be attributed to a substantial loss of high V_MY_ values for both the MS patient and 72 year. The EPWF was substantially higher for the 72 year and the 45 year-MS compared with the healthy 45 year. These findings are consistent with general myelin loss and edema during aging and MS disease progression.

The behavior of the partial volume components around the MS lesion of the 45 year-MS, displayed in the zoomed sections shown in Figure 8, is particularly interesting. The hyper-intensity on the FLAIR image has diffuse edges, making it difficult to estimate the exact volume of the lesion. However, on the V_MY_ image, a clear center, where the myelin has completely vanished, can be observed. At the same location, there is an elevation of the V_EPW_, but this area is larger and decreases toward 0 outwards. On a FLAIR image, no distinction can be made between edema and myelin loss because both processes result in a hyper-intense signal. Using the model, on the other hand, the partial volume images indicate a demyelinated center within a larger area of edema. This example suggests that the model can distinguish between myelin loss and the presence of excess water in edema.

An interesting derivate of the model is the total aqueous content and the corresponding, remaining non-aqueous content. The used sequence cannot resolve the short R_2_ relaxation component and, therefore, the observed PD value will correspond to the visible PD of the medium and long-time components. Using the observed shift in R_1_ and R_2_ the model can predict the presence of the myelin component and, therefore, the true PD value as would be measured at an echo time of 0. The non-aqueous content (Figure 9B) can be attributed to the presence of macromolecules in the brain. From the results, it can be derived that the macromolecular content for the 45 year-MS in the MS lesions was 15–5%, of normal appearing WM approximately 30%, and of normal appearing GM approximately 15%. These results are very similar to the reported values of Mezer et al. (33) and Abbas et al. (34). Our intention is to validate our results further on larger groups of MS patients in future work. Within the possible restrictions of ethical permission, the actual myelin content must be validated by histopathology in combination with the selective staining of individual tissue components.

In Table 2 the MWF is also listed, as directly derived from the model PD values. The definitions of V_MY_ and MWF are not identical; V_MY_ is the estimated MYF of an acquisition voxel based on the effective relaxation properties of that voxel, whereas MWF corresponds to the ratio of observable short-time relaxation (myelin) and medium-time relaxation (cellular) water content. The calculated MWF values are considerably lower than V_MY_ (43% on average). The cause is that MyW only covers a fraction of the total MYV, which also includes the (non-observable) myelin semi-solids. An issue reported by Zhang et al. (35), however, may cause a difference between our observed MWF and the reported MWF values: Using the multi-echo T_2_ relaxation in combination with the NNLS method, the magnetization exchange, responsible for the shift of the medium-time component, is ignored. Such an exchange not only results in a shift of the medium-time component, but is also responsible for a simultaneous decrease in the short-time component. This will lead to a lower observed value for MWF. Studies measuring MWF using multi-exponential T_2_ relaxation indeed reported lower values than our estimated MWF values, such as 7.0–10.1% in WM, 3.6–5.6% in the putamen, and 4.5–4.7% in the thalamus (8, 10, 36–38), compared with our values of 15, 9, and 12%, respectively (Table 3). By contrast, the mcDESPOT approach does account for magnetization exchange and consequently exhibits considerably higher values of MWF. For example, the observed MWF values were as high as 28–30% for WM, 11–13% for the putamen, and 14–15% for the thalamus (13), which are more in the range of our estimated V_MY_ values. In our opinion, this discrepancy is a highly interesting field that must be explored and further understood. A thorough validation study on patients and healthy controls using our method will be the subject of future research.

A limitation of our approach is that the model had to be grossly simplified in order to provide any reasonable results. Each compartment can have very different behavior throughout the brain and with various diseases. Magnetic interaction was reduced to two exchange rates and a number of parameters were fixed to reasonable, but unvalidated values. Adding more degrees of freedom, however, would make it impossible for the model to converge to a solution. The used spatial normalization process resulted in a low resolution of the brain images. This inevitably led to the loss of anatomical detail and smearing of tissue characteristics, which can explain the differences between the values of Table 3 and Tables 4 and 5. For example, voxels that are partially filled with bulk CSF at the periphery of the brain may be seen at low resolution as brain tissue with V_EPW_. Indeed, the spatially normalized brains of the controls in Figure 5 had a relatively high amount of V_EPW_ at 57 mL, whereas all three individual examples in Figure 6 had much lower values. The estimated V_EPW_ for healthy controls in Table 2 was high for the insula, cortical GM, pons, and corpus callosum; the caudate nucleus showed an extreme value for the MS group. All of these structures interface with bulk CSF and, hence, the V_EPW_ likely is lower in reality. Also, GM and WM structures may be blended at this resolution. For the spatially normalized brain images of the healthy controls cortical GM had 15% and WM had 23% V_MY_, whereas the differences between GM and WM for all subjects in Ref. (21), and for the three example subjects, were much more extreme, 2–7% for GM and 26–41% for WM. In Figure 7, V_MY_ peaks can be observed at 5 and 32% for the 45 year (6A), and V_CL_ peaks can be observed at 68 and 92% (6B), which are likely to be centered at GM and WM. A higher resolution of the spatial normalization procedure would likely change the values of Table 3 and make them more similar to those of Tables 4 and 5. Future work will focus on high-resolution spatial normalization in combination with a better definition for the regions of interest to improve the distinction between the various brain structures. Standard, template ROIs have an advantage over (time-consuming and user-dependent) manually placed ROIs, but our data show that the loss of anatomic detail has a large effect on the results.

Another limitation of our method is that the measured V_MY_ properties in Table 1 have large SDs. This is a result of the relatively shallow minimum in the optimization, where a change in one parameter can be compensated for by a change in other parameters. Therefore, our model cannot accurately determine the characteristics of pure V_MY_. The effect of parameter changes in V_MY_ on the calculated grid in Figure 4, however, is relatively small. Brain parenchyma typically has <30% V_MY_, and substantial changes near 100% V_MY_ only have a small effect at lower values. For example, when perturbing R_1,MY_ and PD_MY_ by one SD, the grid points of V_MY_ in Figure 4 changed by <5%, indicating that our model is relatively robust for practical purposes.

All parameters of the model were adapted to 1.5 T spatially normalized data. Because relaxation rates change with field strength, the modeled grid from Figure 4 must be re-optimized for other field strengths. Furthermore, it is important to realize that the partial volumes are measured by observation of magnetic properties of the brain. The fast-relaxing, non-observable MyW has a magnetization exchange with the surrounding cellular water, resulting in an increase in the effective relaxation rate of cellular water in the vicinity. This effect will decrease with distance and, thus, cannot define a hard boundary. Therefore, myelin partial volume in the acquisition voxel reflects the extent of the effect of magnetization exchange in space rather than defining a physical boundary of the myelin sheets. Using this argument, it is not likely that the measured total MYV using this model is identical to the total MYV, which could be measured by summing the volumes of all myelin sheets under a microscope.

For quantitative monitoring of patients in clinical routine, we consider it important that not only the brain volume is monitored. Although this is an important clinical measure, it is only a volumetric measure. It does not reveal any pathological changes in the tissue composition of the brain. Neurological degeneration is related to differences in R_1_, R_2_, and PD and may be characterized by the observation of changes in these values. In this work, an attempt was made to capture the change in quantitative values in a clinically realistic context using the MYF, which is an indirect measure of the myelination degree of the brain, and the EPWF, which is an indirect measure of edema in the brain. Therefore, we believe that BPF, MYF, and EPWF are complementary measures to monitor the quantity and quality of the patient’s brain in relation to intervention or progress of disease or aging.

In conclusion, a model is presented in which each MRI acquisition voxel in the brain is composed of a myelin partial volume, a cellular partial volume, a free water partial volume, and an excess parenchymal water partial volume. The magnetization vector evolution during an MRI quantification sequence was simulated for all partial volume distributions. The parameters of the model were obtained using spatially normalized brain data of a group of healthy control subjects. The differences for a pathological brain were described with myelin loss and the presence of excess parenchymal water. Application of the model showed clear differences between the control group and a spatially normalized MS group, as well as among three individual examples of high-resolution imaging of a healthy middle-aged subject, an elderly control subject, and an MS patient. Using this model, clinically important information, such as the brain volume, degree of myelination, and degree of edema, may be estimated based on an acquisition with a clinically acceptable scan time.

## Author Contributions

MW, ME, and AT contributed to the design, the acquisition, analysis, and interpretation of data for the work; MW, ME, AT, and PL all contributed to writing, reviewing and given final approval of the manuscript.

## Conflict of Interest Statement

The authors declare that the research was conducted in the absence of any commercial or financial relationships that could be construed as a potential conflict of interest.
